# Valor Prognóstico da Troponina T e do Peptídeo Natriurético Tipo B em Pacientes Internados por COVID-19

**DOI:** 10.36660/abc.20200385

**Published:** 2020-10-13

**Authors:** Gustavo Luiz Gouvêa de Almeida, Fabricio Braga, José Kezen Jorge, Gustavo Freitas Nobre, Marcelo Kalichsztein, Paula de Medeiros Pache de Faria, Bruno Bussade, Guilherme Loures Penna, Vitor Oliveira Alves, Marcella Alecrim Pereira, Paula de Castro Gorgulho, Milena Rego dos Santos Espelta de Faria, Luis Eduardo Drumond, Fabrini Batista Soares Carpinete, Ana Carolina Lessa Brandão Neno, Augusto César de Araújo Neno

**Affiliations:** 1 Casa de Saúde São José Rio de JaneiroRJ Brasil Casa de Saúde São José, Rio de Janeiro, RJ – Brasil

**Keywords:** Betacoronavírus, SARS-CoV-2, Pandemia, Biomarcadores, Pacientes Internados, Troponina T, Peptideo Natriurético Tipo B, Doenças Cardiovasculares/complicações

## Abstract

**Fundamento:**

A COVID-19 causa grave acometimento pulmonar, porém o sistema cardiovascular também pode ser afetado por miocardite, insuficiência cardíaca e choque. A elevação de biomarcadores cardíacos tem sido associada a um pior prognóstico.

**Objetivos:**

Avaliar o valor prognóstico da Troponina T (TnT) e do peptídeo natriurético tipo B (BNP) em pacientes internados por Covid-19.

**Métodos:**

Amostra de conveniência de pacientes hospitalizados por COVID-19. Foram coletados dados dos prontuários com o objetivo de avaliar a relação da TnT e o BNP medidos nas primeiras 24h de admissão com o desfecho combinado (DC) óbito ou necessidade de ventilação mecânica. Análise univariada comparou os grupos com e sem DC. Modelo multivariado de Cox foi utilizada para determinar preditores independentes do DC.

**Resultados:**

Avaliamos 183 pacientes (idade=66,8±17 anos, sendo 65,6% do sexo masculino). Tempo de acompanhamento foi de 7 dias (1 a 39 dias). O DC ocorreu em 24% dos pacientes. As medianas de TnT e BNP foram 0,011 e 0,041 ng/dl (p<0,001); 64 e 198 pg/dl (p<0,001) respectivamente para os grupos sem e com DC. Na análise univariada, além de TnT e BNP, idade, presença de doença coronariana, saturação de oxigênio, linfócitos, dímero-D, proteína C reativa titulada (PCR-t) e creatinina, foram diferentes entre os grupos com e sem desfechos. Na análise multivariada *boostraped* apenas TnT (1,12[IC95%1,03-1,47]) e PCR-t (1,04[IC95%1,00-1,10]) foram preditores independentes do DC.

**Conclusão:**

Nas primeiras 24h de admissão, TnT, mas não o BNP, foi marcador independente de mortalidade ou necessidade de ventilação mecânica invasiva. Este dado reforça ainda mais a importância clínica do acometimento cardíaco da COVID-19. (AArq Bras Cardiol. 2020; 115(4):660-666)

## Introdução

O mundo vive atualmente a pandemia de uma doença denominada pela *World Health Organization* (WHO) como COVID-19, causada por um novo coronavírus (SARS-Cov-220). O *International Committee on Taxonomy of Viruses* então denominou o vírus como SARS-CoV-2^1^ (síndrome respiratória aguda severa coronavírus 2). A atual pandemia teve origem na China, em dezembro de 2019, na cidade de Wuhan, capital da província de Hubei. Rapidamente se espalhou globalmente e quando esse artigo foi escrito, já infectou mais de 4,5 milhões de pessoas, causando mais de 300.000 mortes. No Brasil mais de 200 mil pessoas já foram infectadas, sendo 15 mil delas vítimas fatais.

Coronaviroses habitualmente causam doença pulmonar e intestinal aguda, o que faz de seus principais sintomas serem tosse, febre, dispneia, diarreia, náuseas e vômitos. Todavia, desde seu início na China relatos crescentes de seu acometimento do sistema cardiovascular têm alertado a comunidade científica. Elevação de biomarcadores cardíacos, como Troponina T (TnT) e peptídeo natriurético tipo B (BNP) tem sido associada a um pior prognóstico.^2^ Guo et al.,^2^ numa coorte de 187 pacientes hospitalizados na cidade de Wuhan, verificaram que 27,8% dos pacientes apresentaram elevação da TnT e a presença de complicações como necessidade de ventilação mecânica foi maior neste grupo. Os níveis de N-terminal-pro-peptídeo natriurético tipo B (NT-Pro-BNP) tiveram correlação linear positiva significativa com a TnT. Liu et al.,^3^ demonstram que níveis de BNP>100 pg/ml também estiveram associados a um risco maior de complicações em pacientes com COVID-19. Entretanto, ambos os estudos se limitaram a realizar análise univariada dos dados.

Em recente análise de pacientes recuperados de COVID-19, Huang et al.,^4^ demonstrou que a ressonância magnética cardíaca (RMC) foi anormal em 58%. Realce tardio, expressão de fibrose miocárdica, esteve presente em 31% dos pacientes. Porém não houve diferenças nem de TnT nem de BNP entre os grupos com e sem alterações na RMC.

O envolvimento cardíaco na COVID-19 é uma realidade, porém o potencial preditivo dos marcadores cardíacos ainda precisa ser mais bem avaliado.

No presente artigo avaliamos a presença e o impacto dos biomarcadores cardíacos TnT e do BNP, medidos nas primeiras 24h de admissão hospitalar na evolução clínica de pacientes admitidos por COVID-19.

## Métodos

Amostra de conveniência de análise de banco de dados de pacientes internados por COVID-19 em hospital terciário na cidade do Rio de Janeiro. Foram revisados os prontuários médicos de pacientes que preenchiam os critérios de síndrome clínica compatível com COVID-19 pela WHO^5^ e que posteriormente tiveram seu diagnóstico confirmado através de *swab* de nasofaringe pelo método de reação em cadeia da polimerase (PCR) em tempo real. Dados clínicos e laboratoriais foram coletados dessa população. A TnT ultrassensível foi dosada pelo método de eletroquimioluminescência (Elecsys® Troponin T Gen 5 STAT, Laboratório Roche) e seu valor de corte foi de < 0,014 ng/mL e o BNP) pelo método imunoensaio por fluorescência (Triage® BNP; Alere) e seu valor de corte é < 100 pg/mL. Foram ainda analisados sexo, peso, altura, presença de comorbidade (doença coronariana [DAC], doença pulmonar, acidente vascular cerebral, diabetes, hipertensão arterial, doença renal crônica [DRC] e câncer), tempo de sintomas na chegada ao hospital (dias), pressão sistólica (PAS; mmHg), frequência cardíaca (batimentos por minuto) e saturação arterial de oxigênio (%) na admissão, leucócitos totais (células/mm^3^), linfócitos (células/mm^3^), proteína C reativa titulada (PCR-t; mg/dl), creatinina (mg/dl), dímero-D (ng-dl) e ferritina (ng/ml). Todos os parâmetros clínicos e laboratoriais foram obtidos nas primeiras 24 horas da admissão.

O desfecho clínico avaliado foi a combinação de morte por todas as causas ou necessidade de ventilação mecânica (VM).

O estudo foi conduzido de acordo com os padrões da declaração de Helsinque para pesquisa humana. Foi solicitado ao CEP dispensa do termo de consentimento livre e esclarecido, por se tratar de estudo observacional, retrospectivo, de análise de prontuário médico.

### Análise Estatística

As variáveis contínuas foram expressas em média e desvio padrão ou mediana e intervalo interquartil e comparadas pelos testes T-student não-pareado ou U-Mann-Whitney de acordo com a presença ou não de distribuição normal. A presença de distribuição normal foi avaliada pelo teste de Kolmogorov-Smirnov. As variáveis categóricas foram expressas em frequências (%) e comparadas através do teste de Chi-quadrado e exato de Fisher.

Os pacientes foram agrupados em quartis de acordo com os valores de TnT e a evolução dos seus grupos foi comparada através da curva de Kaplan-Meier e a diferença entre os grupos estabelecida pelo teste de log rank.

Análise de sobrevida multivariada de Cox foi desenvolvida com objetivo de identificar preditores independentes de morte e/ou necessidade de VM. Nesses modelos foram incluídas as variáveis com erro alfa menos que 5% na análise univariada. Utilizamos a análise multivariada de sobrevida, pois consideramos mais apropriada para um estudo prognóstico. Para verificar a estabilidade do resultado, e eventuais vieses gerados por *overfitting*, a técnica de *bootstraping* com 1.000 amostra foi empregada.^6^^,^^7^

A significância estatística foi definida por uma probabilidade de erro alfa <5%. A análise estatística foi feita utilizando o programa SPSS (SPSS 22.0 para Windows, IBM SPSS, IL, USA).

## Resultados

Foram analisados 183 pacientes. Tempo mediano de acompanhamento foi de 7 dias (1 a 39 dias). A Tabela 1 descreve as características da população.

**Tabela 1:** Características da população

N	183
Idade (anos)	66,8±17
Peso	80±19
Altura	169±15
Sexo Masculino (%)	65,6
DAC (%)	19,1
Doença Pulmonar (%)	15,8
AVC (%)	4,4
Diabetes (%)	19,7
HAS (%)	53,6
Câncer (%)	9,8
DRC (%)	2,2
Tempo de Sintomas	6(3;8)
PAS	128±19
FC	85±16
SatO_2_	93,6±5,4
Leucócitos	6710(4760;9100)
Linfócitos	1070(740;1400)
PCR-t	9,94(5,48;18,39)
Creatinina	0,98(0,78;1,26)
BNP	84(21;197,5)
TnT	0,011(0,006;0,033)
Dímero D	906(482;1429)
Ferritina	720(378;1303)
Óbitos (%)	15,3
VM (%)	16,9
Óbito e/ou VM (%)	24
Internação em UTI (%)	42,6

DAC: doença arterial coronariana; AVC: acidente vascular cerebral; HAS: hipertensão; DRC: doença renal crônica; PAS: pressão arterial sistólica; FC: frequência cardíaca; SatO_2_: saturação de oxigênio; PCR-t: proteína C reativa titulada; BNP: peptídeo natriurético tipo B; TnT: Troponina T; VM: ventilação mecânica; UTI: unidade de terapia intensiva.

Vinte e oito pacientes morreram e 31 precisaram de VM ao longo do período analisado. O desfecho combinado (DC) (óbito e/ou VM esteve presente em 44 (24%) dos pacientes.

A Tabela 2 demonstra a análise univariada nos grupos com e sem DC. Os pacientes com DC eram mais idosos; tinham maior prevalência de DAC; saturação de oxigênio mais baixa, menor número de linfócitos; PCR-t, creatinina, BNP, TnT e dímero-D mais elevados que o grupo sem desfecho. Essas foram as variáveis incluídas no modelo multivariado de COX cujo resultado está demonstrado na Tabela 3.

**Tabela 2:** Análise Univariada

	Vivo e sem VM	Óbito ou com VM	Valor de p
N	139	44	
Idade (anos)	64±16	75,7±16	<0,001
Peso	82±20	75,5±14	0,116
Altura	169,8±14	168,6±19	0,858
Homem/Mulheres	86/53	34/10	0,061
DAC (%)	14,4	34,1	0,004
Doença Pulmonar (%)	14,4	20,5	0,337
AVC (%)	3,6	6,8	0,401
Diabetes (%)	20,1	18,2	0,775
HAS (%)	51,1	61,4	0,233
Câncer (%)	8,6	13,6	0,311
DRC (%)	1,4	4,5	0,244
Tempo de Sintomas	6(3;8)	4(2,25;7)	0,14
PAS	127,9±19	128,3±21	0,911
FC	85,6±17	87±13	0,405
SatO_2_	94,3±5	91,7±7	0,036
Leucócitos	6510(4715;8905)	7490(5680;10190)	0,083
Linfócitos	1120(832,5;1470)	750(540;1190)	0,001
PCR-t	9,54(4,5325;16,9525)	13,64(7,04;24,74)	0,011
Creatinina	0,92(0,7575;1,0925)	1,3(1,01;1,91)	<0,001
BNP	64,5(16,75;138)	198(45;619)	<0,001
TnT	0,01(0,006;0,017)	0,041(0,012;0,072)	<0,001
Dímero D	741(452,75;1254,75)	1315(776;2200)	<0,001
Ferritina	654(375,5;1204,75)	976(401,5;1543)	0,255

DAC: doença arterial coronariana; AVC: acidente vascular cerebral; HAS: hipertensão; DRC: doença renal crônica; PAS: pressão arterial sistólica; FC: frequência cardíaca; SatO_2_: saturação de oxigênio; PCR-t: proteína C reativa titulada; BNP: peptídeo natriurético tipo B; TnT: Troponina T.

**Tabela 3:** Análise multivariada de Cox com 1.000 *bootstraped*

Variáveis	HR (IC95%)	HR (IC95%) *bootstraped*
Idade (anos)	1,02(0,99-1,04)	1,02(0,97-1,05)
DAC (%)	1,09(0,47-2,53)	1,09(0,36-2,84)
SatO_2_ (%)	0,92(0,87-0,97)	0,92(0,85-1,01)
Linfócitos (cada 100 células/mm^3^)	1,01(0,95-1,07)	1,01(0,87-1,06)
Dímero D (500 Ung/ml)	0,99(0,97-1,01)	0,99(0,92-1,03)
PCR-t (mg/dl)	1,04(1,01-1,08)	1,04(1,00-1,10)
Creatinina (mg/dl)	0,9(0,62-1,3)	0,9(0,55-2,17)
TnT (incremento de 0,014 ng/dl)	1,13(1,05-1,21)	1,12(1,03-1,47)
BNP (incremento de 100 pg/ml)	1,05(0,95-1,15)	1,05(0,81-1,23)

DAC: doença arterial coronariana; SatO_2_: saturação de oxigênio; PCR-t: proteína C reativa titulada; TnT: Troponina T; BNP: peptídeo natriurético tipo B.

Todos os biomarcadores citados foram incluídos na análise multivariada e após a análise de *bootstrap* apenas a TnT e a PCR-t estiveram de forma independente associada ao DC.

**Figura 1:**
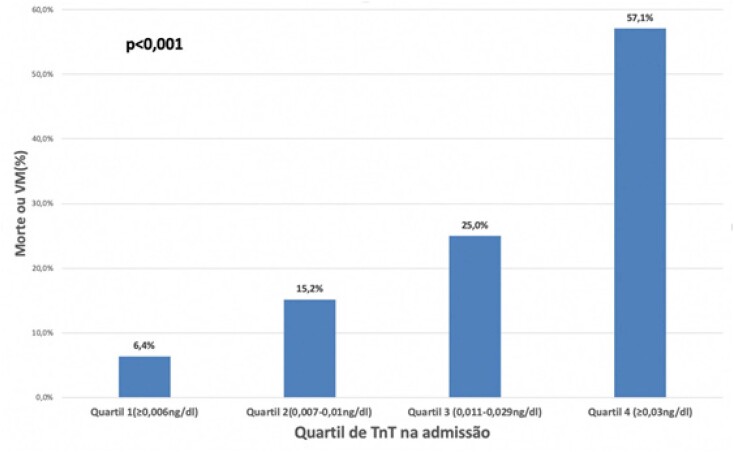
Diferenças de desfecho combinado por quartil de Troponina. VM: ventilação mecânica; TnT: Troponina T.

**Figura 2:**
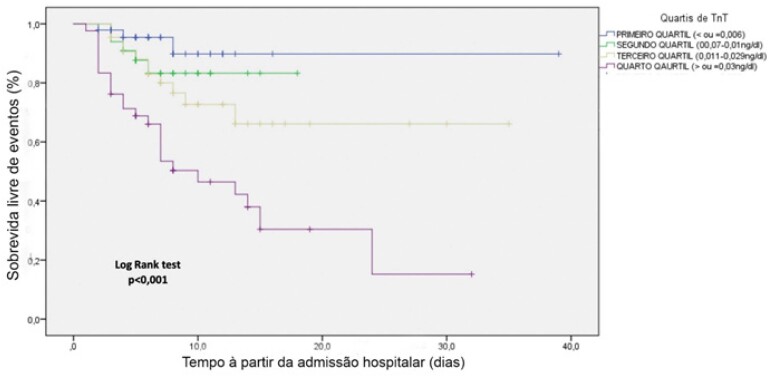
Probabilidade do desfecho combinado ao longo do tempo para cada um dos quartis de Troponina. TnT: troponina T.

A Figura 1 ilustra as diferenças de desfecho combinado por quartil de TnT. A mortalidade mais do que dobra entre Q1 e Q2; e Q3 e Q4, e aumenta mais de 60% entre Q2 e Q3. A Figura 2 mostra a probabilidade do evento ao longo do tempo para cada um dos quartis de TnT. Após 20 dias da admissão, a sobrevida livre de eventos para o Q1 de troponina T (TnT≤0,006ng/dl) foi de 89,8% e para o Q4 (TnT≥0,03ng/dl) foi de 15,2%.

## Discussão

Esse estudo reforça a impressão já levantada por outros autores de que a elevação de TnT, além de prevalente, está associada a evolução para formas graves de COVID-19. A luz dos nossos conhecimentos este é o segundo estudo, primeiro no Brasil, há identificar a TnT como preditor independente de pior prognóstico em pacientes com COVID-19. Shi et al. em coorte chinesa de desenho semelhante, demonstrou que a elevação de troponina na admissão aumentou em 3,41(IC95% 1,62-716) o risco de morte em pacientes com COVID-19.^8^ Nela pacientes com aumento da troponina tiveram uma maior taxa de VM invasiva frente aos que não tiveram elevação da troponina (18 de 82 [22,0%] vs. 14 de 334 [4,2%]; p< 0,001). Além disso a mortalidade também foi maior naqueles com injúria miocárdica frente aos sem injúria (42 de 82 [51,2%] vs. 15 de 334 [4,5%]; p< 0,001). Todavia, epidemia de outras doenças virais como a Dengue na China tiveram prevalência e prognóstico de miocardite bem diferentes que o Brasil, e outros países.^9^^,^^10^ Nosso trabalho demonstra que este não parece ser o caso da COVID-19, onde, em populações ocidentais e orientais, a prevalência de injúria miocárdica é prevalente e associada a pior prognóstico. Dentre os desfechos desfavoráveis encontram-se insuficiência cardíaca, arritmias, VM e morte.^11^

Entre os mecanismos propostos para a agressão miocárdica causada pelo SARS-CoV-2, temos principalmente a chamada “tempestade de citocinas” que é desencadeada por um desequilíbrio nas respostas celulares dos linfócitos T helper Tipo 1 e Tipo 2. A Interleucina-6 (IL-6) é uma das que se elevam como resultado desse desequilíbrio celular, sendo inclusive um marcador de mortalidade já identificado. Essas citocinas agridem o miocárdio, causando elevação da troponina e disfunção cardíaca.^12^ Uma metanálise de quatro estudos chineses publicados como correspondência, envolvendo 341 pacientes foi publicada recentemente.^13^ A prevalência de elevação de troponina (acima do percentil 99%) foi de 8 a 12%, e seus valores foram significativamente mais elevados em pacientes com formas mais graves de COVID-19. Portanto, a monitorização da troponina pode ajudar a identificar um subgrupo com maior chance de um curso clínico pior.

Um importante achado no estudo de Guo et al.^5^ foi que a elevação da troponina foi um marcador mais forte para mortalidade do que a presença de doença cardiovascular (DCV) prévia. Pacientes com histórico de DCV, mas com troponina normal tiveram mortalidade menor que aqueles sem história de DCV, mas que elevaram troponina na internação. Além disso, tanto a TnT quanto NT-pro-BNP aumentaram de forma significativa ao longo da internação naqueles que evoluíram para óbito e isto não foi observado naqueles que sobreviveram.

Na nossa coorte ficou muito bem demonstrada a relação da elevação da TnT e o DC de morte ou VM, chegando ao ponto de que mais da metade dos pacientes no último quartil de troponina (>0,03ng/dL) tiveram evolução desfavorável. Isso pode ajudar de forma prática a identificar na admissão aqueles pacientes com maior risco intra-hospitalar de pior curso clínico.

Quanto ao BNP/NT-pro-NBP, alguns estudos também sugerem ser um marcador prognóstico importante. Os possíveis mecanismos para elevação do BNP na infecção por SARS-Cov-2 vão desde a elevação secundária a agressão inflamatória do miocárdio (tempestade de citocinas), já descrita, que resulta em disfunção cardíaca e aumento das pressões de enchimento ventricular, até mesmo a agressão direta do cardiomiócito pelo vírus pelo sítio de ligação da enzima conversora da angiotensina 2 e pela hipoxemia miocárdica induzida pela injúria pulmonar aguda. O primeiro estudo que mostrou que o NT-Pro-BNP é um marcador de mortalidade foi publicado por Gao et al.,^14^ 54 pacientes com disfunção respiratória importante (frequência respiratória ≥30/min ou SatO_2_ ≤93% ou relação entre pressão arterial de O_2_/fração inspirada de oxigênio ≤ 300 mmHg). Pacientes com NT-proBNP> 88,64 pg/mL mostraram uma sobrevida cumulativa no segmento de 15 dias significativamente menor do que aqueles com valores abaixo desse valor. Na nossa coorte apesar de um preditor de risco na análise univariada, o BNP não se mostrou um marcador independente de risco quando utilizado o modelo multivariado. Isso pode ser explicado por um efeito de colinearidade entre TnT e BNP, uma vez que já foi demonstrado grande correlação entre estes marcadores na COVID-19.

A proteína C reativa titulada além da TnT, também esteve associada independentemente a um pior prognóstico em nossa coorte. De fato, outros trabalhos já vêm sinalizando a correlação que existe entre a PCR-t e a severidade da infecção por COVID-19,^15^^,^^16^ o que suporta os achados do nosso trabalho.

O presente estudo mostrou a relação entre a elevação da TnT e o risco para óbito ou necessidade de VM. Em contrapartida, a elevação do BNP, apesar de na análise univariada ter se mostrado um fator de risco para o DC de VM ou óbito, não se mostrou um preditor independente na nossa amostra. De fato, recente artigo de revisão de Costa et al.,^17^ estabeleceu fluxograma de abordagem cardiológica de pacientes com COVID-19 e a troponina foi o único marcador laboratorial sugerido para definir internação em unidade de terapia intensiva, independentemente da presença de história de DCV.

### Limitações

Dados de eletrocardiograma (ECG) e ecocardiograma (ECO) não foram incluídos na análise. Isso porque menos de 70% dos pacientes da amostra tem esses dados. O paciente COVID-19 é um grande consumidor de recursos hospitalares notoriamente EPI e por isso esses exames só são solicitados quando estritamente necessários e indicados. Isso também valeu para não avaliarmos os biomarcadores como variáveis contínuas ao longo do tempo. Não utilizamos coletas seriadas de rotina desses biomarcadores. Cada entrada no leito de isolamento para coleta de biomarcadores ou de outros exames, salvo estritamente necessário, aumenta os custos, a utilização de EPIs e o risco para a equipe de saúde. Portanto, incluí-los (ECG, ECO e biomarcadores coletados seriadamente) exigiria uma estratégia de tratamento de dados faltantes que, na nossa opinião, comprometeria a análise.

Outra limitação é de que com muitos preditores na análise univariada e um número de desfechos relativamente pequenos para o tamanho amostral, a técnica de *bootstrap* não elimina a possibilidade de *overfitting*.

## Conclusão

A TnT, mas não o BNP, foi um marcador independente de risco para mortalidade ou necessidade de VM invasiva em pacientes hospitalizados por COVID-19. Estes dados reforçam ainda mais a utilização deste biomarcador na estratificação de risco dos pacientes com COVID-19.
