# Supplementary material for: Valor Prognóstico da Troponina T e do Peptídeo Natriurético Tipo B em Pacientes Internados por COVID-19
Source: Arq Bras Cardiol. 2020 Oct 13;115(4):660–6. [Article in Portuguese] doi: 10.36660/abc.20200385 (PMC8386964; doi:10.36660/abc.20200385)
Supplement: Translation

# Translation: Prognostic Value of Troponin-T and B-Type Natriuretic Peptide in Patients Hospitalized for COVID-19

**Authors:** Gustavo Luiz Gouvêa de Almeida, Fabricio Braga, José Kezen Jorge, Gustavo Freitas Nobre, Marcelo Kalichsztein, Paula de Medeiros Pache de Faria, Bruno Bussade, Guilherme Loures Penna, Vitor Oliveira Alves, Marcella Alecrim Pereira, Paula de Castro Gorgulho, Milena Rego dos Santos Espelta de Faria, Luis Eduardo Drumond, Fabrini Batista Soares Carpinete, Ana Carolina Lessa Brandão Neno, Augusto César de Araújo Neno

**Affiliations:** 1 Rio de JaneiroRJ Brazil Casa de Saúde São José, Rio de Janeiro, RJ – Brazil

**Keywords:** Betacoronavirus, SARS-CoV-2, Pandemics, Biomarkers, Inpatients, Troponin T, Natriuretic Peptide, B Type, Cardiovascular Diseases/complications

## Abstract

**Background:**

COVID-19 causes severe pulmonary involvement, but the cardiovascular system can also be affected by myocarditis, heart failure and shock. The increase in cardiac biomarkers has been associated with a worse prognosis.

**Objectives:**

To evaluate the prognostic value of Troponin-T (TNT) and natriuretic peptide (BNP) in patients hospitalized for Covid-19.

**Methods:**

This was a convenience sample of patients hospitalized for COVID-19. Data were collected from medical records to assess the association of TnT and BNP measured in the first 24 hours of hospital admission with the combined outcome (CO) of death or need for mechanical ventilation. Univariate analysis was used to compare the groups with and without the CO. Cox's multivariate model was used to determine independent predictors of the CO.

**Results:**

We evaluated 183 patients (age = 66.8±17 years, 65.6% of which were males). The time of follow-up was 7 days (range 1 to 39 days). The CO occurred in 24% of the patients. The median troponin-T and BNP levels were 0.011 and 0.041ng/dL (p <0.001); 64 and 198 pg/dL (p <0.001), respectively, for the groups without and with the CO. In the univariate analysis, in addition to TnT and BNP, age, presence of coronary disease, oxygen saturation, lymphocytes, D-dimer, t-CRP and creatinine, were different between groups with and without outcomes. In the bootstrap multivariate analysis, only TnT (1.12 [95% CI 1.03-1.47]) and t-CRP (1.04 [95% CI 1.00-1.10]) were independent predictors of the CO.

**Conclusion:**

In the first 24h of admission, TnT, but not BNP, was an independent marker of mortality or need for invasive mechanical ventilation. This finding further reinforces the clinical importance of cardiac involvement in COVID-19. (Arq Bras Cardiol. 2020; 115(4):660-666)

## Introduction

The world is currently experiencing the pandemic of a disease called COVID-19 by the World Health Organization (WHO), caused by a new coronavirus (SARS-Cov-2). The International Committee on Taxonomy of Viruses then called the virus SARS-CoV-2^1^ (severe acute respiratory syndrome coronavirus-2). The current pandemic originated in China in December 2019 in the city of Wuhan, capital of the Hubei province. It quickly spread globally and by the time this article was written, it has already infected more than 4.5 million people, causing more than 300,000 deaths. In Brazil, more than 200,000 people have already been infected, of which 15,000 died due to COVID-19.

Coronaviruses usually cause acute lung and intestinal disease, of which its main symptoms are cough, fever, dyspnea, diarrhea, nausea and vomiting. However, since its appearance in China, there have been growing reports of its cardiovascular system involvement, which have alerted the scientific community. Elevated cardiac biomarkers, such as Troponin-T (TnT) and brain natriuretic peptide (BNP) has been associated with a worse prognosis^2^. Guo et al.,^2^ in a cohort of 187 hospitalized patients in the city of Wuhan, found that 27.8% of the patients had an increase in TnT and the presence of complications, such as the need for mechanical ventilation, was higher in this group. The levels of N-terminal-pro-brain natriuretic peptide (NT-Pro-BNP) had a significant positive linear correlation with TnT. Liu et al.^3^ showed that BNP levels >100pg/mL were also associated with a higher risk of complications in patients with COVID-19. However, both studies were limited to performing the univariate analysis of data.

In a recent analysis of patients that recovered from COVID-19, Huang et al.^4^ demonstrated that cardiac magnetic resonance (CMR) imaging was abnormal in 58%. Late enhancement and myocardial fibrosis expression were present in 31% of patients. However, there were no differences in either TnT or BNP levels between groups with and without changes in CMR imaging.

Cardiac involvement in COVID-19 is a reality, but the predictive potential of cardiac markers still needs to be better assessed.

In the present article, we evaluated the presence and impact of cardiac biomarkers TnT and BNP, measured within the first 24 hours of hospital admission on the clinical evolution of patients admitted for COVID-19.

## Methods

This was a convenience sample obtained from the database analysis of patients admitted for COVID-19 in a tertiary hospital in the city of Rio de Janeiro, Brazil. The medical records of patients who met the criteria for a clinical syndrome compatible with COVID-19 by the WHO^5^ and who later had their diagnosis confirmed by a nasopharyngeal swab using the real-time polymerase chain reaction (RT-PCR) method were reviewed. Clinical and laboratory data were collected from this population. The ultrasensitive TnT was measured using the electrochemiluminescence method (Elecsys^®^ Troponin T Gen 5 STAT, Roche Laboratory) and its cutoff value was <0.014ng/mL, whereas the BNP was measured by the fluorescence immunoassay method (Triage^®^ BNP; Alere) and its cutoff value was <100 pg/mL.

Gender, weight, height, presence of comorbidity (coronary artery disease [CAD], pulmonary disease, stroke, diabetes, arterial hypertension, chronic kidney disease [CKD] and cancer), time of symptom onset on arrival at the hospital (days), systolic blood pressure (SBP; mmHg), heart rate (beats per minute) and arterial oxygen saturation (%) on hospital admission, total leukocytes (cells/mm^3^), lymphocytes (cells / mm3), titrated C-reactive protein (t-CRP; mg/dL), Creatinine (mg/dL), D-dimer (ng-dL) and Ferritin (ng/mL) were also assessed. All clinical and laboratory parameters were obtained within the first 24 hours of hospital admission.

The assessed clinical outcome was the combination of death from all causes or the need for mechanical ventilation (MV).

The study was carried out according to the standards of the Helsinki declaration for human research. The need for the Free and Informed Consent was waived by the Research Ethics Committee, as it is an observational, retrospective study, performed through the analysis of medical records.

### Statistical Analysis

The continuous variables were expressed as mean and standard deviation or median and interquartile range and compared by unpaired Student's *t* test or Mann-Whitney-U test according to the presence or not of normal distribution. The presence of normal distribution was assessed by the Kolmogorov-Smirnov test. Categorical variables were expressed as frequencies (%) and compared using the Chi-square test and Fisher's exact test.

The patients were grouped in quartiles according to the troponin-T values and the evolution of their groups was compared using the Kaplan-Meier curve, whereas the difference between groups was established by the log rank test. Cox multivariate survival analysis was developed aiming to identify independent predictors of death and or the need for mechanical ventilation. Variables with an alpha error < 5% in the univariate analysis were included in these models. The multivariate analysis of survival was used, as it was considered more appropriate for a prognostic study.

To verify the stability of the result, and any biases generated by overfitting, the bootstrapping technique with 1,000 samples was used.^6^^,^^7^

Statistical significance was defined by an alpha error probability <5%. Statistical analysis was performed using the SPSS program (SPSS 22.0 for Windows, IBM SPSS, IL, USA).

## Results

A total of 183 patients were analyzed. The median follow-up time was 7 days (1 to 39 days). Table 1 describes the characteristics of the population.

**Table 1:** Characteristics of the population

N	183
Age (years)	66.8±17
Weight	80±19
Height	169±15
Male gender (%)	65.6
CAD (%)	19.1
Pulmonary disease (%)	15.8
Stroke (%)	4.4
Diabetes (%)	19.7
SAH (%)	53.6
Cancer (%)	9.8
CKD (%)	2.2
Time of symptom onset	6(3;8)
SBP	128±19
HR	85±16
SatO_2_	93.6±5.4
Leukocytes	6710(4760;9100)
Lymphocytes	1070(740;1400)
CRP	9.94(5.48;18.39)
Creatinine	0.98(0.78;1.26)
BNP	84(21;197.5)
TnT	0.011(0.006;0.033)
D-Dimer	906(482;1429)
Ferritin	720(378;1303)
Deaths (%)	15.3
Mechanical Ventilation – MV (%)	16.9
Death and/or MV (%)	24
Admission at ICU (%)	42.6

CAD: coronary artery disease; SAH: systemic arterial hypertension; CKD: chronic kidney disease; SBP: systolic blood pressure; HR: heart rate; SatO_2_: oxygen saturation; t-CRP: C-reactive protein; BNP: B-Type Natriuretic Peptide; TnT: Troponin-T; MV: mechanical ventilation; ICU: intensive care unit.

Twenty-eight patients died and 31 required mechanical ventilation during the analyzed period. The combined outcome (death and/or mechanical ventilation was present in 44 (24%) of the patients.

Table 2 shows the univariate analysis in the groups with and without the combined outcome. The patients with a combined outcome were older; had a higher prevalence of CAD; lower oxygen saturation, fewer lymphocytes; and higher levels of t-CRP, creatinine, BNP, TnT and D-dimer than the group without the outcome. These were the variables included in the COX multivariate model, of which results are shown in table 3.

**Table 2:** Univariate Analysis

	Alive without MV	Death or with MV	p-value
N	139	44	
Age (years)	64±16	75.7±16	<0.001
Weight	82±20	75.5±14	0.116
Height	169.8±14	168.6±19	0.858
Men/Women	86/53	34/10	0.061
CAD (%)	14.4	34.1	0.004
Pulmonary disease (%)	14.4	20.5	0.337
Stroke (%)	3.6	6.8	0.401
Diabetes (%)	20.1	18.2	0.775
SAH (%)	51.1	61.4	0.233
Cancer (%)	8.6	13.6	0.311
CKD (%)	1.4	4.5	0.244
Time of symptom onset	6(3;8)	4(2.25;7)	0.14
SBP	127.9±19	128.3±21	0.911
HR	85.6±17	87±13	0.405
SatO_2_	94.3±5	91.7±7	0.036
Leukocytes	6510(4715;8905)	7490(5680;10190)	0.083
Lymphocytes	1120(832.5;1470)	750(540;1190)	0.001
CRP	9.54(4.5325;16.9525)	13.64(7.04;24.74)	0.011
Creatinine	0.92(0.7575;1.0925)	1.3(1.01;1.91)	<0.001
BNP	64.5(16.75;138)	198(45;619)	<0.001
TnT	0.01(0.006;0.017)	0.041(0.012;0.072)	<0.001
D-Dimer	741(452.75;1254.75)	1315(776;2200)	<0.001
Ferritin	654(375.5;1204.75)	976(401.5;1543)	0.255

CAD: coronary artery disease; SAH: systemic arterial hypertension; CKD: chronic kidney disease; SBP: systolic blood pressure; HR: heart rate; SatO_2_: oxygen saturation; TnT: Troponin-T; MV: mechanical ventilation; ICU: intensive care unit; CRP: C-reactive protein.

**Table 3:** Cox multivariate analysis with 1000 bootstrapped

Variables	HR (95%CI)	HR (95%CI) bootstrapped
Age (years)	1,02(0,99-1,04)	1,02(0,97-1,05)
CAD (%)	1,09(0,47-2,53)	1,09(0,36-2,84)
SatO_2_ (%)	0,92(0,87-0,97)	0,92(0,85-1,01)
Lymphocytes (each 100 cells/mm^3^)	1,01(0,95-1,07)	1,01(0,87-1,06)
D-Dimer (500Ung/mL)	0,99(0,97-1,01)	0,99(0,92-1,03)
CRP (mg/dL)	1,04(1,01-1,08)	1,04(1,00-1,10)
Creatinine (mg/dL)	0,9(0,62-1,3)	0,9(0,55-2,17)
TnT (increment of 0.014ng/dL)	1,13(1,05-1,21)	1,12(1,03-1,47)
BNP (increment of 100pg/mL)	1,05(0,95-1,15)	1,05(0,81-1,23)

CAD: coronary artery disease; SatO_2:_ oxygen saturation; CRP: C-reactive protein; BNP: B-Type Natriuretic Peptide; TnT: Troponin-T.

All mentioned biomarkers were included in the multivariate analysis and after the bootstrap analysis, only TnT and t-CRP were independently associated with the combined outcome.

Figure 1 illustrates the differences in the combined outcome per TnT quartile. Mortality more than doubles between Q1 and Q2; and between Q3 and Q4 and increases by more than 60% between Q2 and Q3. Figure 2 shows the probability of the event over time for each of the TnT quartiles. After 20 days of admission, the event-free survival rate for the first interquartil (Q1) of troponin T (TnT≤0.006ng/dl) was 89.8% and for the last interquartil (Q4) (TnT≥0.03ng/dl) was 15.2%.

**Figure 1:** Differences in combined outcome by Troponin quartile. MV: mechanical ventilation; TnT: Troponin-T.

**Figure 2:** Probability of the combined outcome over time for each of the Troponin quartiles. TnT: Troponin-T.

## Discussion

This study reinforces the idea previosuly raised by other authors that the increase in TnT, in addition to being prevalent, is associated with the evolution to severe forms of COVID-19. To the best of our knowledge, this is the second study, the first in Brazil, to identify TnT as an independent predictor of a worse prognosis in patients with COVID-19. Shi et al., studying a Chinese cohort with a similar design, demonstrated that increased troponin levels at hospital admission increased the risk of death in patients with COVID-19 by 3.41-fold (95%CI, 1.62-716). In this cohort, patients with increased troponin levels had a higher rate of invasive mechanical ventilation compared to those who did not have an increase in troponin (18 of 82 [22.0%] *vs*. 14 of 334 [4.2%]; p <0.001). Additionally, mortality was also higher in those with myocardial injury compared to those without injury (42 of 82 [51.2%] vs 15 of 334 [4.5%]; p <0.001). However, the epidemic of other viral diseases such as dengue in China showed very different prevalence rates and prognosis of myocarditis than Brazil, and other countries.^9^^,^^10^ Our study shows that this does not seem to be the case for COVID-19, where, in both western and eastern populations, the prevalence of myocardial injury is prevalent and associated with a worse prognosis. Among the unfavorable outcomes are heart failure, arrhythmias, mechanical ventilation and death.^11^

Among the mechanisms proposed for myocardial injury caused by SARS-CoV-2, there is mainly the so-called “cytokine storm”, which is triggered by an imbalance in the cell responses of Type-1 and Type-2 T-helper lymphocytes. Interleukin-6 (IL-6) is a cytokine that increases as a result of this cell imbalance and it is an already identified marker of mortality. These cytokines attack the myocardium, causing elevation in troponin levels and cardiac dysfunction.^12^

A meta-analysis of 4 Chinese studies involving 341 patients was recently published as correspondence.^13^ The prevalence of troponin elevation (above the 99^th^ percentile) ranged from 8 to 12%, and its values were significantly higher in patients with more severe forms of COVID-19. Therefore, the monitoring of troponin levels may help to identify a subgroup with a greater chance of a worse clinical course.

An important finding in the study by Guo et al.^5^ was that the increase in troponin levels was a stronger marker for mortality than the presence of previous cardiovascular disease (CVD). Patients with a history of CVD, but with normal troponin levels had lower mortality rates than those without a history of CVD, but who had increased troponin levels at hospital admission. Moreover, both TnT and NT-pro-BNP increased significantly during hospitalization in those who died, and this increase was not observed in those who survived.

In our cohort, the association between troponin-T elevation and the combined outcome of death or MV was very well demonstrated, to the point that more than half of the patients in the last quartile of troponin (> 0.03ng/dL) had an unfavorable evolution. This can constitute a practical way to identify those patients with the highest in-hospital risk of a worse clinical course on admission.

As for the BNP/NT-pro-BNP, some studies also suggest that it is an important prognostic marker. Possible mechanisms for the increase in BNP levels in the presence of SARS-Cov-2 infection range from the previously described elevation secondary to inflammatory myocardial injury (cytokine storm), which results in cardiac dysfunction and increased ventricular filling pressures, to the direct injury to the cardiomyocyte by the virus through the angiotensin-converting enzyme-2 binding site and due to the myocardial hypoxemia induced by acute lung injury. The first study that showed that NT-Pro-BNP is a marker of mortality was published by Gao et al.,^14^ evaluating 54 patients with significant respiratory dysfunction (respiratory rate ≥30/min or Sat O_2_≤93% or ratio of Partial Pressure Arterial Oxygen and Fraction of Inspired Oxygen ≤ 300mmHg). Patients with NT-proBNP >88.64 pg/mL showed a significantly lower cumulative survival during the 15-day follow-up than those with levels below this value. In our cohort, despite being a risk predictor in the univariate analysis, BNP was not an independent risk marker when the multivariate model was used. This fact can be explained by a collinearity effect between TnT and BNP, as a great correlation has been demonstrated between these markers in COVID-19.

In addition to TnT, the titrated C-reactive protein was also independently associated with a worse prognosis in our cohort. In fact, other studies have already indicated the presence of a correlation between t-CRP and the severity of infection by Covid-19,^15^^,^^16^ which supports the findings of our study.

The present study showed an association between elevated TnT levels and the risk of death or need for MV. In contrast, the increase in BNP levels, although it was shown to be a risk factor for the combined outcome of MV or death in the univariate analysis, was not shown to be an independent predictor in our sample. In fact, a recent review article by Costa et al.^17^ established a flowchart for the cardiological approach of patients with COVID-19 and troponin was the only laboratory marker suggested to define admission in the intensive care unit, regardless of the presence of a history of cardiovascular disease.

### Limitations

Electrocardiogram and echocardiogram data were not included in the analysis, as less than 70% of patients in the sample had these data available. The patient with COVID-19 is a major consumer of hospital resources, notably PPE, which is why these tests are only requested when strictly necessary and indicated. This was also the reason for not evaluating biomarkers as continuous variables over time. We did not use routine serial collections of these biomarkers. Every time a healthcare professional enters the isolation area for the collection of biomarkers or other tests, unless strictly necessary, results in increased costs, the use of PPE and a risk for the entire health team. Therefore, including them (ECG, ECHO and serial collections of biomarkers) would require a strategy for treating the missing data, which in our opinion would compromise the analysis.

Another limitation is that with many predictors in the univariate analysis and a number of relatively small outcomes for the sample size, the bootstrap technique does not eliminate the possibility of overfitting.

## Conclusion

Troponin T, but not BNP, was an independent risk marker for mortality or need for invasive mechanical ventilation in patients hospitalized for COVID-19. These data further reinforce the use of this biomarker in the risk stratification of patients with COVID-19.
